# Research Advances of Porous Polyimide—Based Composites with Low Dielectric Constant

**DOI:** 10.3390/polym15163341

**Published:** 2023-08-08

**Authors:** Zhenjiang Pang, Hengchao Sun, Yan Guo, Jun Du, Liang Li, Qiuyang Li, Junzhong Yang, Jijun Zhang, Weiguo Wu, Sen Yang

**Affiliations:** 1 Beijing Smart–Chip Microelectronics Technology Co., Ltd., Beijing 100192, China; pangzhenjiang@sgchip.sgcc.com.cn (Z.P.); guoyan5@sgchip.sgcc.com.cn (Y.G.); liliang@sgchip.sgcc.com.cn (L.L.); 2 China Electric Power Research Institute, No. 15 Xiaoying East Road, Beijing 100192, China; liqyang@126.com; 3 State Grid Taizhou Power Supply Company, Taizhou 225300, China; 13337788026@189.cn (J.Y.); 13914522336@139.com (J.Z.); wuweiguo@js.sgcc.com.cn (W.W.); naziiyong@163.com (S.Y.)

**Keywords:** dielectric constant, polyimide, porous materials, composite

## Abstract

With the burgeoning of the microelectronics industry, in order to improve the transmission speed between chips in large-scale integrated circuits to meet the demands of high integration, it is necessary for interlayer insulation materials to possess a lower dielectric constant (k). Polyimide (PI) has been widely used as interlayer insulation materials for large-scale integrated circuits, and the exploration on reducing their dielectric constant has attracted extensive attention in recent years. In this work, porous PI-based composites with a low dielectric constant are mainly reviewed. The application of porous SiO_2_, graphene derivatives, polyoxometalates, polyhedral oligomeric silsesquioxane and hyperbranched polysiloxane in reducing the dielectric constant of PI is emphatically introduced. The key technical problems and challenges in the current research of porous polyimide materials are summarized, and the development prospect of low k polyimide is also expounded.

## 1. Introduction

With the advancement of society, electronic components are evolving toward the direction of thin, light and small, so that miniaturization of large-scale integrated chips becomes a development tendency. However, several problems such as multiplication of circuit power loss, increase of reaction time, short circuit and so on also come along. Equation (1) presents the relationship between signal transmission speed, delay time and dielectric constant (k) that is strongly frequency dependent in parts of the frequency spectrum [1,2]:

(1) $$T=RC=2\rho {\varepsilon \varepsilon }_{0}[\frac{4L^{2}}{P^{2}}+\frac{L^{2}}{D^{2}}]$$

where *T* is the signal delay time, *R* is the resistance, *C* is the capacitance, *ρ* is the specific resistance of the conductor, *ε* is the dielectric constant of the insulating material, *ε*_0_ is the dielectric constant of vacuum, *L* is the length of the conductor, *D* is the thickness of the conductor and *P* is the distance between two conducting lines. In order to cut down the signal delay during transmission and meet the demand of high integration in integrated circuits, the dielectric constant of metal interlayer insulation materials should be as low as possible, which can diminish the parasitic capacitance effect between metal wires. Nowadays, traditional SiO_2_ materials (k ≈ 4) cannot follow the further development of integrated circuits [3,4]. Therefore, the exploration of new low-k materials has become an inevitable tendency.

At present, common inorganic low k materials include zeolite, dry gel, porous silicon carbide, etc. [5,6], but their thin film mechanical strength and actual processing characteristics are far inferior to low k polymer materials. Meanwhile, as a metal interlayer dielectric material, in addition to meeting the requirement of low dielectric performance, a higher breakdown field strength is beneficial for extending the service life of electronic devices when operated at high frequency/high voltage. Furthermore, dielectric materials should also possess the merits like low moisture absorption, good mechanical properties and high thermal stability [1,7].

**Figure 1:**
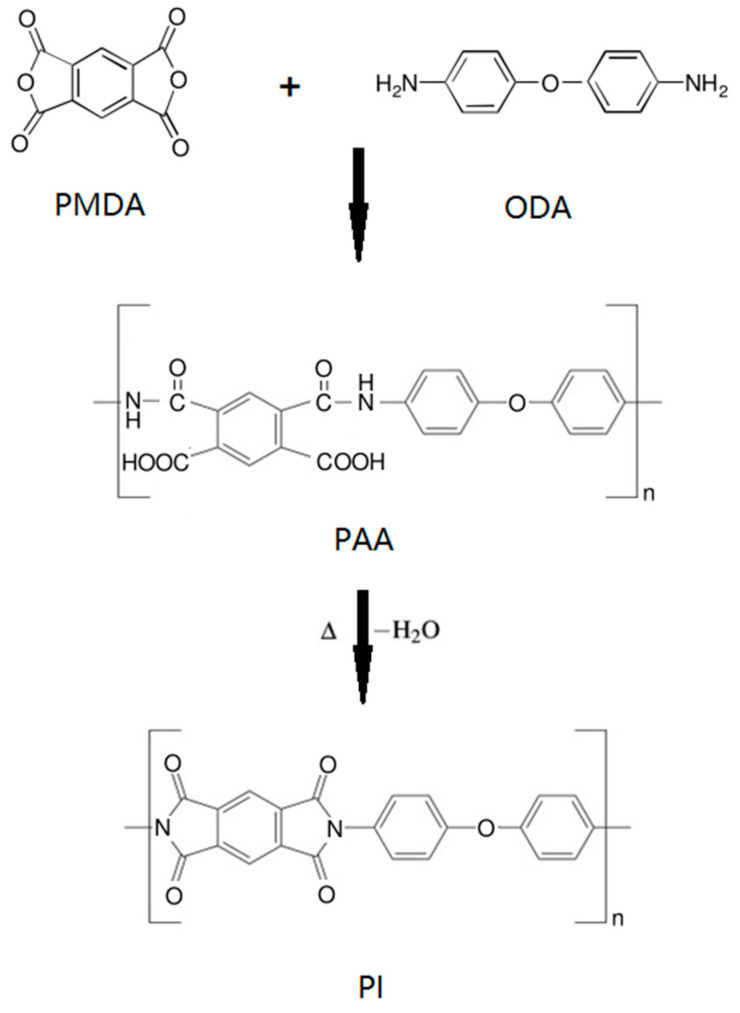
Reaction scheme for the preparation of a typical PI.

Polyimide (PI) is a kind of semi-aromatic or aromatic polymer consisting of imide rings typically fused to phenyls. Figure 1 shows the preparation of a typical PI from the thermal imidization of polyamic acid (PAA) that can be obtained from pyromellitic dianhydride (PMDA) and 4,4′-oxydianiline (ODA). Because of its good mechanical properties, thermal stability and insulation properties, PI has been widely used in aerospace, automobile, electronics and other fields [8,9,10]. Li et al. [11] synthesized a PAA solution by ternary copolymerization of 3,3′,4,4′-biphenyl tetracarboxylic dianhydride (BPDA), p-phenylenediamine (PDA) and 2,2′-bis (trifluoromethyl)-4,4′-diamino biphenyl (TFDB), and obtained PI films with uniform pores through the processes such as thermal imidization, casting and film laying. Due to the rigid structure of TFDB and the presence of –CF_3_, the dielectric relaxation time was high, and the dielectric constant decreased from 3.42 to 2.96 at 1 MHz. Moreover, the PI films exhibited a low dielectric loss of 1.6 × 10^−2^ in the whole frequency range. Mehdipour-Ataei et al. [12] prepared nano PI foams with a pore size of 5–20 nm by using PMDA and common diamines as polymerization monomers. The experimental results showed that the dielectric constant of nano PI foam was 2.46–2.73, and it still maintained good stability at 370 °C. However, the structure of these intrinsic porous PIs depends on monomers with strong rigidity and a twisted molecular structure, so there exist more requirements for the selection of monomers, and their dielectric constants are still high. Therefore, researchers have taken various measures to diminish the dielectric constant of PI.

This work mainly summarizes the progress on low k PI-based materials in recent years, including the introduction of their preparation, the elucidation of the influencing mechanisms of dielectric properties, the description of the existing problems and challenges as well as their development prospects.

## 2. Methods for Reducing the Dielectric Constant of PI

By now, there are few reports on reducing the dielectric constant of PI through physical methods, mainly including plasma surface treatment [13], the reprecipitation method [14], electron/proton irradiation [15] and gamma ray irradiation [16]. Moreover, Krause et al. [17] added supercritical CO_2_ into two commercial PAA and two synthetic PAA solutions, and gradually released pressure during the process of high-temperature thermal imidization to make CO_2_ overflow, thus creating nanopores. It was found that the higher bubble temperature caused the smaller pore size and lower dielectric constant. When the volume content of pores in the PI film reached 40 vol.%, the dielectric constant was as low as 1.7 at 1 kHz. Although this method produces nanofoam with uniform dispersion and controllable size during the preparation of PI, no new chemical pollution occurs. However, the application of this method in industrial production is limited by some problems such as imperfect foam forming theory and complex process control.

Chemical methods are commonly used to improve the dielectric properties of PI, which mainly include the following strategies: The first is introducing substituents with lower molar polarizability on the PI chain, such as fluorine (F) atoms [18,19]. The introduction of fluorinated groups can diminish the symmetry or regularity of polymer monomers, and improve the free volume of the system, thereby reducing the dielectric constant of the polymer. Simone et al. [20] explored a series of high fluorine PIs with different C–F bonds, and the polymer monomer with the highest fluorine content contained 8 fluorine atoms. The experimental results showed that the dielectric constant of high fluorine PI arrived at 2.6~2.7 at 1 kHz. Dong et al. [21] designed three novel diamines containing pyridine and –C(CF_3_)_2_ groups for polymerization with 2,2′-bis (3,4-dicarboxyphenyl) hexafluoropropane dianhydride (6FDA) to obtain fluorinated PI (FPI) containing pyridine and –C(CF_3_)_2_ groups. The FPI film manifested a low dielectric constant (2.36~2.52) at 1 MHz, and still maintained good mechanical properties. Wang et al. [22] fabricated a series of PI fibers with low dielectric constants via a two-step wet-spinning method by incorporating 2,2′-bis(trifluoromethyl)-4,4′-diaminobiphenyl (TFMB) into rigid polymer backbones of BPDA/PDA. The dielectric constant of PI fibers was substantially cut down from 3.0 to 2.48 at 10 GHz after the introduction of –CF_3_ groups. Li et al. [23] manufactured a series of porous low k PI films through the nonsolvent-induced phase separation of a soluble FPI. By adjusting the mass ratio of the binary solvent used to dissolve the PI, the structure could be regulated accurately and the PI film with a porosity of 48% displayed a low dielectric constant of 2.48 at 10 GHz. By studying the influence of the fluorine group on the aggregation structure and properties of PI fibers, it was found that the improved free volume was the crucial factor responsible for a low dielectric constant. However, the introduction of fluorine atoms often leads to a decrease in the glass transition temperature (Tg), mechanical strength and bonding strength of PI [24].

The second is to introduce hollow structures such as pores or gaps in the structure to lower the overall dielectric constant of the material [25,26,27]. The construction of the pore structure is to introduce air. As the dielectric constant of air is about 1, by increasing the porosity of the system and decreasing the density of the material, the number of polarized molecules per unit volume is reduced, thereby suppressing the dielectric constant of the material. The methods of directly introducing pore structures in PI mainly include thermal degradation into pores [28,29] and etching [30,31,32].

**Figure 2:**
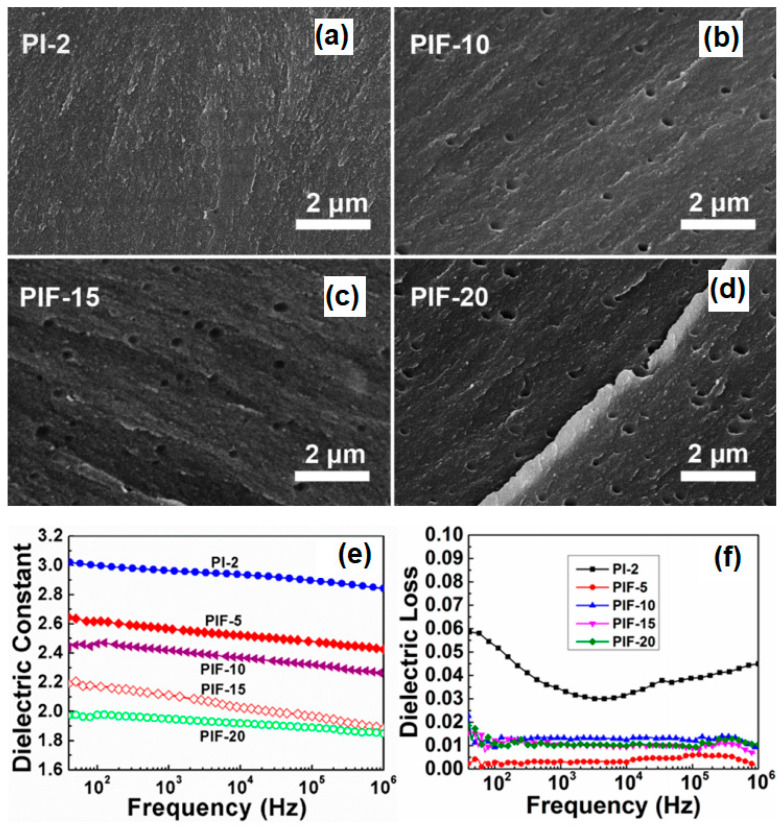
SEM images of PI film and porous films by removing the PEG amounts of (**a**) 0 wt.% (PI-2), (**b**) 10 wt.% (PIF-10), (**c**) 15 wt.% (PIF-15) and (**d**) 20 wt.% (PIF-20). Dielectric constant (**e**) and dielectric loss (**f**) of porous PI films with the removal of various PEG amounts. Reprinted with permission from Ref. [35].

In the thermal degradation method, the thermally unstable polymer is dispersed into the PAA, and the unstable components are degraded into low molecular products by imidization, so that the nanopores can be introduced into the PI matrix by the diffusion and escape of low molecular products in the film. Wang et al. [28] treated the soluble FPI in ozone, and then carried out thermally induced graft copolymerization with acrylic acid. By decomposing the side chain of polyacrylic acid, nanoscale pores were introduced into the PI matrix. When the grafting rate was 1.67, the porosity was 8% and the dielectric constant of the film decreased from 3.1 to 1.9 at 1 MHz. Lv et al. [33] used 2,2′-bis (trifluoromethyl) benzidine (TFDB) and 6FDA as polymerization monomers, and successfully prepared FPI matrix membranes containing nanopores by adding thermally unstable polymer polyethylene glycol (PEG) after chemical imidization and heat treatment. With the increased PEG content, the porosity of the PI film increased, and the dielectric constant correspondingly decreased from 2.80 to 2.12 at 1 MHz. Within the temperature range of −150~150 °C, its dielectric constant was kept stable at around 2.46. Jin et al. [24] incorporated Pluronic block copolymers into PAA and formed a spherical particle stacking structure through self-assembly. After thermal imidization and solvent evaporation, the Pluronic block copolymer template was removed, forming a porous PI structure with a diameter of less than 50 nm, and a minimum dielectric constant of 1.92 at 1 MHz. Ding et al. [34] reported the synthesis of porous PI aerogel films through a partial pre-imidization process via drying and freeze-drying under ambient pressure. The effect of pre-imidization degree on the dielectric properties of porous PI films was studied and a low dielectric constant (minimum 1.414 at 1 MHz) was achieved. Lv et al. [35] studied a series of ultralow dielectric porous PI films with low water absorption by incorporating the flexible polydimethylsiloxane (PDMS) segments and rigid adamantane groups into PI chains and thermolysis of PEG added to the PI matrix. The porous PI films showed uniform and regularly shaped nanopores with an average diameter of 237 ± 19 nm, as shown in Figure 2a–d. A significant suppression in the dielectric constant from 2.84 to 1.85 at 1 MHz was observed with the increased PEG amount removal to 20 wt.% and the average dielectric loss was 0.012 at 1 MHz, as presented in Figure 2e,f. The adamantane groups and PDMS segments contributed to the lower polarizability, and the bulky adamantane groups and nanopores simultaneously offered enhanced free volumes, which caused improved dielectric performance. However, although the method of thermal degradation to create pores is simple to operate, several problems still exist, such as prolonged thermal decomposition procedures, low porosity, uncontrollable pore size and incomplete decomposition of external components that affect the uniformity of the film. In addition, during the degradation process, it is easy to cause pore collapse to form through-holes, leading to enhanced hygroscopicity and increased dielectric constant.

**Figure 3:**
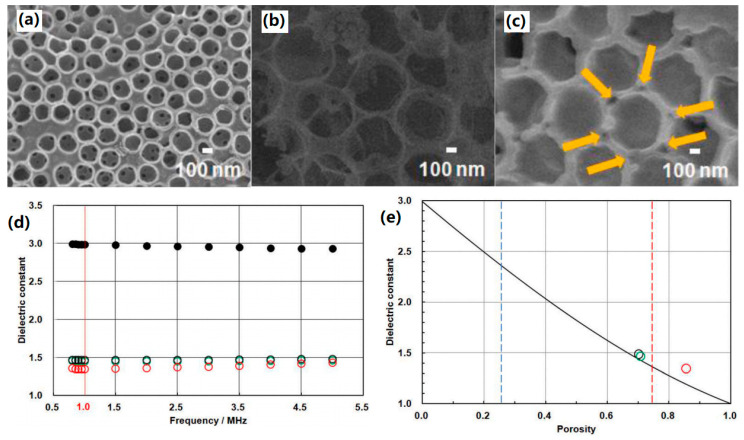
SEM images of porous PI thin films. (**a**) Sample PPI-M (template: Sample M), (**b**) Sample PPI-L (template: Sample L) and (**c**) Sample PPI-SL (template: the mixture of Sample S and Sample L, and the volume fraction of Sample S is 0.206). (**d**) Dielectric constants of various porous PI thin films. Red open circles: Sample PPI-SL; green open circles: Sample PPI-M; black open circles: Sample PPI-L; black closed circles: non-porous PI thin films. (**e**) Plots of dielectric constants vs. porosity. Red, green and black open circles are Sample PPI-SL, Sample PPI-M and Sample PPI-L, respectively. Reprinted with permission from Ref. [37].

The principle of constructing porous PI films by chemical solvent etching is to use PI as a continuous phase and dissolve or etch the dispersed nanofillers using a chemical solvent to form an air-filled PI film. Zhang et al. [32] obtained porous PI films by etching silica nanospheres with hydrofluoric acid (HF) using 6FDA and oxydimethyl aniline as polymerization monomers by the sol–gel method. When the volume fraction of pores was 10.1%, the dielectric constant reached 1.9 at 1 MHz. Jiang et al. [30] used the same process to prepare nanoporous PI films with a pore size of 20–120 nm and found that with the increase of porosity, the minimum dielectric constant of the film could reach 1.84 at 1 MHz. Li et al. [36] employed the breath figure process to prepare microporous PI films via solution casting for direct synthesis of PI film and solvent treatment upon pre-shaped PI film for in situ pore formation. By optimizing the pore structure of the PI film, the dielectric constant was cut down from 3.7 for non-porous film to 1.7 for porous film at 1 M Hz, and the dielectric loss was kept lower than 0.01 over a wide frequency range. Kourakata et al. [37] fabricated porous PI thin films by using silica microparticles with different particle sizes as a template and HF as etchant (Sample S: 42 nm, Sample M: 268 nm, Sample L: 603 nm), and the porous structures could be observed clearly by scanning electron microscopy (SEM) images, as displayed in Figure 3a–c. The introduction of a porous structure remarkably suppressed the dielectric constant of the PI thin films (Figure 3d) and by adjusting the porosity using silica microparticles with different sizes, the dielectric constant was further reduced to the lowest value of 1.35 at 1 M Hz for PPI-SL by using Samples S and L as templates, as shown in Figure 3e. Notably, the utilization of chemical solvents for etching into pores generally involves strong acids, so there exists a certain degree of danger during the operation. In addition, the same as thermal degradation for pore formation, if the introduced filler is not etched or completely dissolved, unnecessary impurities will be introduced, thereby affecting the overall performance of the dielectric material.

Therefore, currently, the widely used method is to combine PI with porous nanoparticles to build PI-based porous composites [38,39]. For example, Qiu et al. [40] reported PI composites formed by PI microspheres with a size of 31–33 μm as fillers and PI composed of PMDA and ODA as the matrix. For 10–50% PI microspheres in the PI composite, the dielectric constant was significantly reduced to 2.26–2.48 (1 MHz) with a dielectric loss of 0.00663–0.00857 (1 MHz), which greatly improved the dielectric properties of the original PI. This composite method can form pores with a skeleton structure in the PI matrix, which not only brings air with a low dielectric constant into the PI matrix but also sustains the pore structure in the form of a skeleton, effectively avoiding pore collapse. Moreover, this method does not bring about low molecular volatiles, without solvent pollution, and importantly, it can effectively reduce some defects, and realize regulation of nanopore size. Up to now, the commonly used low k fillers include porous SiO_2_, graphene derivatives, polyoxometalates (POMs), polyhedral oligomeric silsesquioxane (POSS) and hyperbranched polysiloxane (HBPSi), etc. We will detailedly introduce these low k PI-based porous composite materials in the following sections.

## 3. Porous SiO_2_/PI Composite Material

SiO_2_ is not only a traditional interlayer insulating material for integrated circuits but is also a common inorganic filler for composite materials. It shows the characteristics of high temperature resistance, low thermal expansion coefficient and good chemical stability. Due to the small intrinsic dielectric constant of SiO_2_, if the dual effect of introducing pores is added, the incorporation of SiO_2_ materials with pore structures (mesoporous particles, hollow spheres, hollow tubes, etc.) can more effectively diminish the dielectric properties of PI [41].

### 3.1. Mesoporous SiO_2_/PI Composite Material

Due to the ordered and regular spatial structure of mesoporous SiO_2_, it has attracted widespread attention in many applications such as molecular sieves, catalysts, adsorbents, etc. The dielectric constant of mesoporous SiO_2_ thin film ranges from 1.42 to 2.1, which is smaller than those of most inorganic fillers [42]. Lin et al. [43] reported that SBA-15 and SBA-16 mesoporous SiO_2_ modified with 3-aminotriethoxysilane were added into a PAA solution for in situ polymerization and then subjected to thermal imidization treatment to obtain mesoporous SiO_2_/PI films. It was found that when the content of SBA-15 reached 3 wt.% and 7 wt.%, the dielectric constant of PI decreased from 3.34 to 2.73 and 2.61 at 1 MHz, respectively. Furthermore, the dielectric constant of PI with SBA-16 was lower than that of PI with SBA-15, owing to the smaller pore size and more uniform distribution of SBA-16. Notably, due to the fact that the addition of surface-modified mesoporous SiO_2_ can enhance the interfacial interaction between them and the PI matrix, the thermal stability and dynamic mechanical properties of mesoporous SiO_2_/PI thin films were also improved. Lee et al. [44] prepared mesoporous SiO_2_ with a diameter of 80~140 nm using a hydrothermal synthesis method. After fully mixing mesoporous SiO_2_ with polyamic acid triethylamine salt solution, the porous membrane was obtained by thermal imidization. When the mass fraction of mesoporous SiO_2_ loading was 5%, the dielectric constant of the composite material decreased to 2.45 at 1 kHz, and when the content of mesoporous SiO_2_ increased, the dielectric constant showed a trend of first decreasing and then increasing. Notably, as the content of mesoporous SiO_2_ increased, particle aggregation became heavier, resulting in an increase in the interface area and polarization between the matrix and filler particles, which should be responsible for the elevated dielectric constant of PI composite materials. In addition, at room temperature, the composite films with a high mesoporous SiO_2_ content displayed stronger water absorption, which exerted a significant impact on their dielectric properties, also resulting in an increase in their dielectric constant. Huang et al. [45] prepared fluorinated silica by using KMnO_4_ to etch the silica microspheres into mesoporous structures and grafting 1H,2H,2H,2H-perfluorodecyltriethoxysilane onto the surface of the hollow silica, and further constructed fluorinated silica/PI composite material. It was found that the fluorinated silica could lower the dielectric constant and reduce the hygroscopicity of the composite film. Even at a very low content of 2 wt.%, the dielectric constant of the composite was significantly cut down to 2.61 at 10 MHz without sacrificing thermal stability, which was about 26.7% lower than that of pure PI film. Moreover, the dielectric loss gradually increased with the increased SiO_2_ content due to the higher intrinsic dielectric loss of SiO_2_.

Min et al. [46] added alkylation- and amination-treated SBA-15 into a PAA solution, respectively, followed by imidization treatment to obtain mesoporous SiO_2_/PI films. Their dielectric, mechanical and thermal properties were studied and the results showed that the addition of amino-treated SBA-15 could diminish the dielectric constant of PI to 2.6 at 1 MHz and improve its mechanical properties and thermal stability. Although mesoporous SiO_2_ has the advantages in pore structure, there still exists a drawback of organic–inorganic phase separation when combined with PI materials. To overcome this drawback, Mathews et al. [47] mixed SBA-15 with water-soluble aliphatic PAA ammonium salt and then subjected it to imidization treatment to construct mesoporous SiO_2_/PI films. When the content of SBA-15 reached 7 wt.%, the dielectric constant of the composite film decreased to 2.50 at 1 MHz.

### 3.2. SiO_2_ Hollow Tube/PI Composite Material

In addition to mesoporous SiO_2_, SiO_2_ hollow tube is also a typical porous SiO_2_ structure. Zhang et al. [48] prepared SiO_2_ hollow tubes by self-assembly using tartaric acid templates and hydrolysis of tetraethoxysilane (TEOS) and mixed them with a PAA solution under ultrasonic stirring. After thermal imidization treatment, SiO_2_ hollow tube/PI composite films were obtained. When the mass fraction of SiO_2_ hollow tubes was less than 3%, the dielectric constant decreased with the increase of SiO_2_ content, reaching a minimum of 2.9 at 1 MHz. However, when the mass fraction was more than 10%, the dielectric constant of 3.0 was higher than that of pure PI thin films. This indicates that a linear increase in the proportion of pores does not guarantee a linear decrease in the dielectric constant of the composite material, and the addition of excessive silica fillers can actually cause an elevation in the dielectric constant of PI. Moreover, it can also give rise to a decrease in the mechanical strength, insulation performance and hydrophobicity of the material, resulting in a decrease in the overall performance of the PI [49]. Yudin et al. [50] synthesized SiO_2_ hollow tubes [Mg_3_Si_2_O_5_(OH)_4_] using magnesium and SiO_2_ under hydrothermal conditions. Then, the PAA solution was added into a suspension of N-methylpyrrolidone (NMP) dissolved in hollow tubes for polymerization reaction, and then subjected to thermal imidization treatment to obtain SiO_2_/PI composite films. When the amount of hollow tube was 10 wt.%, the dielectric constant of the composite film decreased to 2.2 at 1 kHz. In addition, as the content of hollow tubes increased, the mechanical properties of the composite film were also improved. Chien et al. [51] fabricated organically modified hollow SiO_2_ nanofibers (HSNFs) via effectively combining electrospinning, high-temperature calcination and chemical modification using 3-aminopropyl(triethoxysilane) (APTEOS). The HSNFs served as a core to construct HSNF/PI composites using two-step polymerization, and the composites manifested low dielectric constants of 2.58 and 2.53 at 28 and 38 GHz, respectively, due to the increase of free volume with the presence of HSNF.

### 3.3. SiO_2_ Hollow Sphere/PI Composite Material

**Figure 4:**
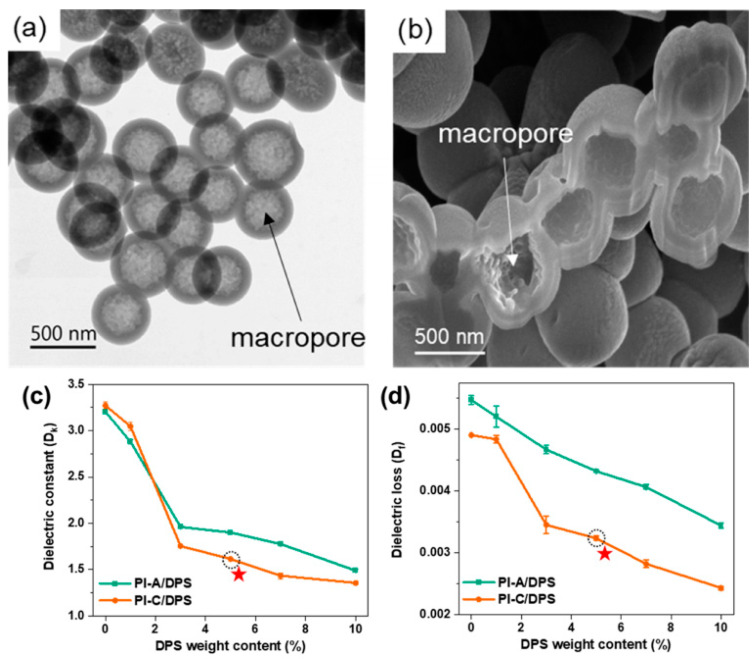
(**a**) TEM and (**b**) cross-sectional SEM images of MPS. (**c**) Dielectric constant and (**d**) dielectric loss of PI-A/DPS and PI-C/DPS composite films with different DPS contents at 28 GHz. PI-A and PI-C represent the PI derived from amine- and carboxyl-terminated PAA precursors, respectively. Reprinted with permission from Ref. [54].

Zhou et al. [52] obtained SiO_2_ hollow spheres with the classical “core shell” hollow structure by the sol–gel method. Then, SiO_2_ hollow spheres/PI porous composite films were built by in situ copolymerization with comonomers such as pyromellitic dianhydride and 4,4′-diaminodiphenyl ether. When the mass fraction of SiO_2_ hollow spheres was 10%, its dielectric constant decreased from 3.41 for pure PI to 2.09 at 1 kHz. However, when the mass fraction of SiO_2_ hollow spheres increased to 13% and 15%, their dielectric constant actually increased. This is mainly due to the fact that as the loading amount of SiO_2_ hollow spheres increases, their dispersibility deteriorates and the interaction with the PI matrix becomes weak. Lee et al. [53] prepared SiO_2_ hollow spheres with a diameter of 200 nm using polystyrene (PS) as a template by the sol–gel method and then mixed them with PAA to obtain SiO_2_ hollow spheres/PI composite films after amination treatment. When the content of SiO_2_ hollow spheres reached 5 wt.%, the dielectric constant of the composite film decreased to 2.24 at 1 kHz while improving its thermal stability. Kim et al. [54] employed a “one-step” water-based emulsion template method to synthesize the microporous silica nanospheres (MPS), as displayed in Figure 4a,b, and dispersed poly(vinylpyrrolidone) functionalized MPS into a PAA solution to create the dual-porous silica nanoparticles (DPS)/PI composite after thermally activated imidization reaction in which the MPS were in situ converted into the DPS with macro- and microporous structures. It was observed from Figure 4c,d that with the addition of 5 wt.% DPS, the dielectric constant and dielectric loss were 1.62 and 0.003 at 28 GHz, respectively, due to the increase in air voids with the incorporation of DPS.

## 4. Graphene Derivative/PI Composite Materials

Graphene is a two-dimensional carbon material that is exfoliated from graphite material and constructed by the hybridization of single-layer carbon atoms through sp^2^ to form a honeycomb-like hexagonal lattice. The unique structure of graphene endows it with good mechanical, electrical, optical and thermal properties. As representatives of graphene derivatives, graphene oxide (GO), reduced graphene oxide (rGO) and fluorinated graphene (FG) have attracted much attention for their application as effective collaborators to reduce the dielectric properties of PI [55].

### 4.1. GO/PI Composite Materials

**Figure 5:**
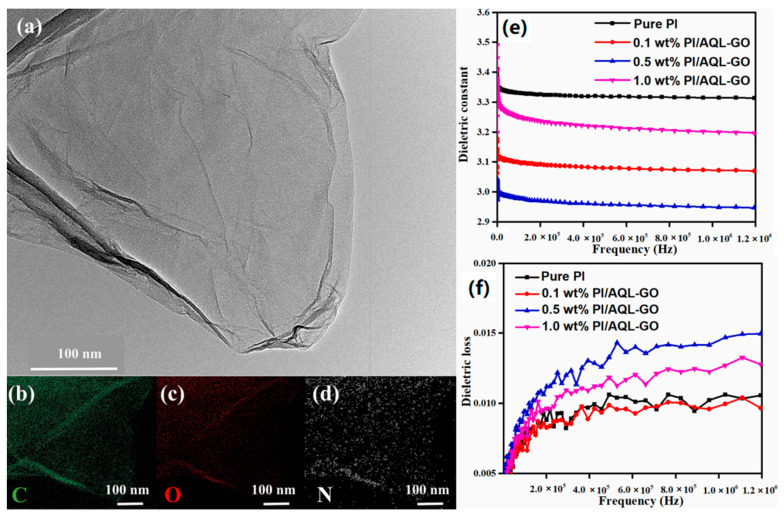
(**a**) TEM image and elemental mappings of (**b**) C, (**c**) O, (**d**) N of AQL-GO. (**e**) Dielectric constant and (**f**) dielectric loss of pure PI, 0.1 wt.%, 0.5 wt.% and 1.0 wt.% PI/AQL-GO composite films. Reprinted with permission from Ref. [60].

Wang et al. [56] grafted ODA onto GO and functionalized it to obtain ODA-GO. Then, ODA-GO/PI composite films were synthesized through in situ polymerization, where GO offered a versatile starting platform by grafting PAA at the reactive sites of GO nanosheets. The tensile strength and tensile modulus of elasticity of the composite film were high, and the dielectric constant decreased with increasing GO content and reached a low value of 2.0 at 1 GHz for 5 wt.% GO concentration in the precursor. Liao et al. [57] first grafted octa(aminophenyl) silsesquioxane (OAPS) onto GO to prepare OAPS-GO with a two-dimensional porous structure. Then, OAPS-GO/PI composite films were synthesized through in situ polymerization. When the content of OAPS-GO reached 3 wt.%, the dielectric constant of the composite film decreased to 1.9 at 1 GHz, and meanwhile, its mechanical properties were significantly improved. Chen et al. [58] grafted GO onto photosensitive PI through a photocrosslinking process to synthesize GO/PI composite films. When the GO content was 0.5 wt.% and the UV irradiation time was 900 s, the dielectric constant and dielectric loss of the composite film reached the lowest, which were 2.58 and 0.026 at 10 MHz, respectively, due to the confined electronic polarization caused by the crosslinked structure. Han et al. [59] fabricated a sandwich-type porous GO/PI film in which GO contained porous FPI film located on both sides of a flat PI film via a facile breath figure method using flat PI film and GO contained FPI organic solution as substrate and casting solution, respectively. The results showed that with the addition of 0.5 wt.% GO/dimethyldioctadecylammonium bromide (DODA), the lowest dielectric constant of 2.21 at 10 MHz was achieved. The suppression in dielectric constant compared with PI film was probably caused by the interaction between GO/DODA and chains of FPI, which could restrict the movement of dipolar moieties and suppress the relaxation of dipoles. Wang et al. [60] prepared aminoquinoline (AQL)-functionalized graphene oxide (GO), as shown in Figure 5a–d, and constructed GO/PI composites by a nucleophilic attack of AQL and chaotic packing of the polymer chain. It was found from Figure 5e,f that the GO/PI displayed a low dielectric constant of 2.96 at 1 MHz with the addition of 0.5 wt.% GO and a low dielectric loss (<0.016). Moreover, the GO/PI possessed good mechanical properties and high thermal stability.

### 4.2. rGO/PI Composite Materials

**Figure 6:**
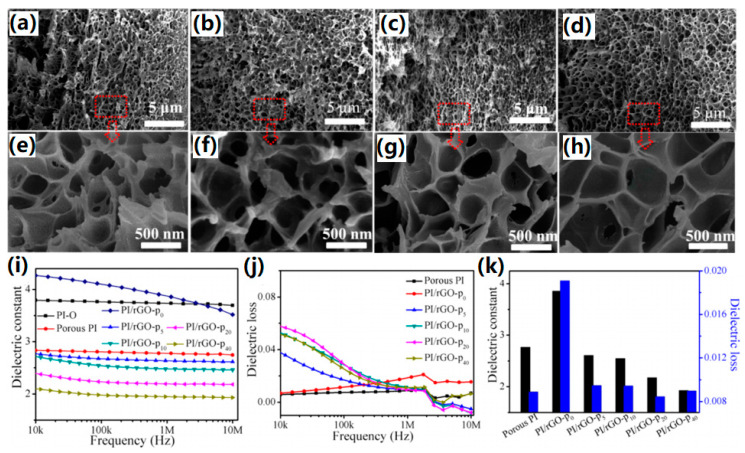
(**a**–**d**) Cross section SEM images of PI/rGO-p_x_ (x = 0, 10, 20, 40, 60, 80, 100) films adjusted by the mass ratios of PEG to PAAC at 5, 10, 20 and 40 wt.%, respectively. (**e**–**h**) Cross sections of the enlarged area in (**a**–**d**). (**i**) Dielectric constant and (**j**) dielectric loss of PI and PI/rGO-p_x_ films vs. frequency. (**k**) Column diagrams of dielectric constant and dielectric loss of PI and PI/rGO-p_x_ films at 1.0 MHz. Reprinted with permission from Ref. [61].

Chen et al. [61] fabricated porous rGO/PI hybrid films by using them as pore-forming agent by mixing the polyamide acid carboxylate (PAAC), GO and PEG, and subsequently heating PAAC/GO/PEG hybrid films. As shown in Figure 6, by regulating the pore structure via adjusting the mass ratio of PEG to PAAC/GO, the obtained rGO/PI films exhibited an ultralow dielectric constant as low as 1.93 when the PEG content reached 40 wt.% and a comparable dielectric loss at 1 MHz with pure PI.

### 4.3. FG/PI Composite Materials

Wang et al. [62] first fluorinated the sponge graphene to obtain fluorinated sponge graphene, and then synthesized FG/PI composite films through in situ polymerization. It was found that the fluorine/carbon ratio, width of band gap and sheet-size of graphene played the important roles in determining the dielectric properties of the composites. When the content of FG was 1 wt.%, the dielectric constant of the composite film decreased to 2.1 at 1 MHz without sacrificing their mechanical properties and thermal stability. Pu et al. [63] put different mass ratios of FG derived from the exfoliation of fluorinated graphite into the PI precursor containing fluorinated blocks to prepare FG/PI composite films via in situ polymerization. It was found that when the FG content reached 0.5 wt.%, the dielectric constant decreased to 1.77 (1 kHz), much lower than that of pure FPI (2.92 at 1 kHz), and also a dielectric loss of 0.005 at 1 kHz was obtained. Zhang et al. [64] employed FG/PAA with different mass ratios as the precursor to construct the FG/PI composite films by phase inversion. When the content of FG was 0.5 wt.%, the dielectric performance of the FG/PI film was the best with a dielectric constant of 1.56 (10 kHz) due to the optimum effective porosity and overall polarization effect. The same group [65] fabricated a novel sandwich structure using an FG/PI composite film as a dense outer surface layer and porous PI film as the middle layer. Such an architecture improved the mechanical and dielectric breakdown performance compared to that of porous PI, and a dielectric constant of 1.92 and a dielectric loss of less than 0.003 were achieved.

## 5. Polyoxometalate/PI Composite Materials

POM is a class of inorganic metal oxide cluster compounds with special properties and structures, which are formed by the polycondensation of transition metal elements M (Mo, W, W, Nb, Ta, etc.) with MO_x_ (usually x is 6) as the unit. POM has attracted more and more attention due to its nanoporous structure, controllable porosity and simple synthesis method. Adding porous POM nanoparticles to the PI matrix is expected to diminish the dielectric constant of composite films. However, the compatibility between POM and PI is poor, so that surface modification is required to realize bulk filling. Tan et al. [66] used a silane coupling agent to organically modify polyoxometalates (SiW_11_–CH=CH_2_) and added them into the PAA solution. Finally, POM/PI composite films were prepared by thermal imidization treatment. The modification of SiW_11_ with vinyltrimethoxysilane caused better dispersion of the particles in the PI matrix, which improved thermal–mechanical and dielectric properties without severely affecting the thermal stability of the PI matrix. When the content of POM arrived at 20 wt.%, the dielectric constant of the composite film declined to 2.05 at 1 MHz. In the composite, the rigid and bulky SiW_11_ unit of SiW_11_–CH=CH_2_ tends to exclude the close contact with surrounding polymer matrix chains, creating a large free volume. The large free volumes and the intrinsic pores of SiW_11_ particles are mainly responsible for the decreased dielectric constant of the PI composites.

## 6. Polyhedral Oligomeric Silsesquioxane/PI Composite Materials

POSS is an organic/inorganic hybrid molecule with a nanocage structure constructed by an inorganic core composed of Si and O elements and surrounded organic groups. Its molecular formula is Si_8_O_12_, which shows a large specific surface area and good skeleton stability. The presence of peripheral organic groups can enhance the interfacial compatibility between POSS and the polymer matrix [67]. The presence of a POSS hollow structure can introduce nanoscale pores into the polymer matrix, improve the free volume of the system and reduce the polarity, achieving the goal of reducing the dielectric constant of composite materials. In addition, the inorganic core of POSS manifests high heat resistance and oxidation resistance, which makes it an ideal material for preparing PI with a low dielectric constant [68].

Leu et al. [69] used 6FDA and hydroxylated diamine as comonomers, and the chlorine-terminated POSS reacted with the hydroxyl groups on the diamine monomer to obtain POSS/PI composite films. The POSS molecules in the amorphous PI retained a nanoporous crystal structure but constructed an additional ordered architecture due to microphase separation. By adjusting the amount of POSS molecules, the dielectric constant of the 35 mol% POSS/PI composite film decreased to 2.4 at 1 MHz, while maintaining certain thermal and mechanical strengths. Wahab et al. [70] utilized allyl alcohol to modify the surface of POSS and obtained hydroxyl terminated POSS, which was added to the PAA solution and finally subjected to thermal imidization to obtain POSS/PI composite films. Because of the intermolecular hydrogen bond between the hydroxyl group on POSS and the PMDA-ODA main chain of PI, POSS could be uniformly dispersed in the polymer matrix under ultrasonic treatment and maintain a certain stability. When the mass fraction of POSS was 10%, the dielectric constant of the composite membrane could be reduced to 2.53. Ye et al. [71] synthesized a reactive fluorine POSS derivative-octakis(dimethylsiloxane hexafluoropropyl glycidyl ether) silsesquioxane by using allyl 1,1,2,3,3-hexafluoropropenyl ether, showing a unique porous cubic structure. The hydrogen bonding effect in the POSS/PI system restricts the movement of molecular chains, improving the heat resistance of the composite material while further increasing the free volume of the system. When the mass fraction of POSS was 15%, the dielectric constant of the system was as low as 2.30 at 100 kHz. The same group [72] further optimized the experimental conditions and cut the dielectric constant of POSS/PI down to 2.12 at 100 kHz by blending the PI precursor with a fluorinated POSS derivative, octakis(dimethylsiloxyhexafluoropropyl) silsesquioxane.

**Figure 7:**
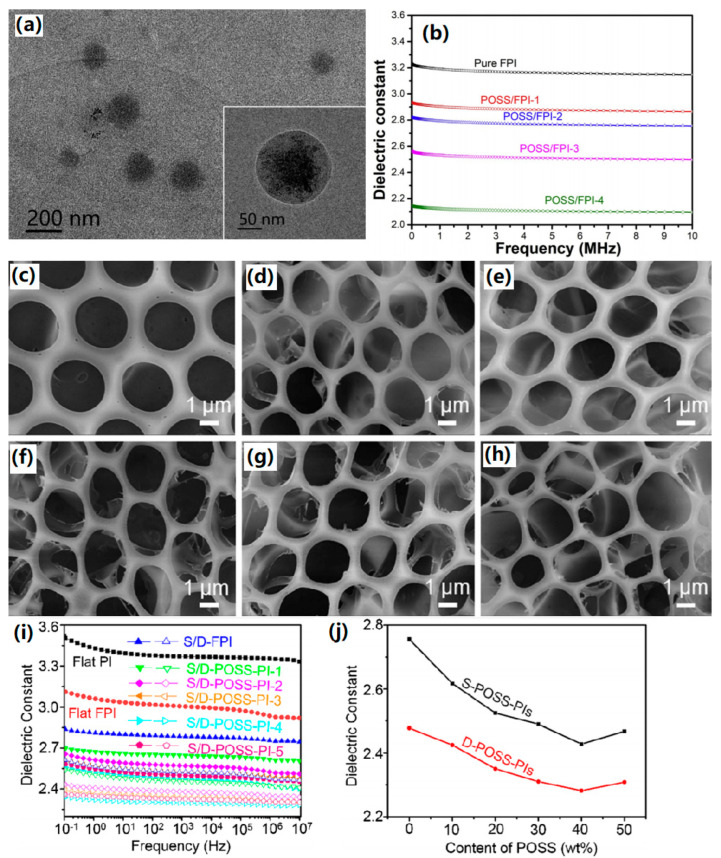
(**a**) TEM images of POSS aggregates. (**b**) Dielectric constants of pure FPI and POSS/FPI composite films. Reprinted with permission from Ref. [76]. SEM images of (**c**) D-FPI and D-POSS-PIs containing different content of POSS: (**d**) 10%, (**e**) 20%, (**f**) 30%, (**g**) 40% and (**h**) 50%. (**i**) Dielectric constants of different samples and (**j**) dielectric constant dependence on the content of POSS. Reprinted with permission from Ref. [80].

Furthermore, Lee et al. [73] grafted polyethylene oxide (PEO) onto POSS via platinum (Pt) catalysis to obtain PEO-POSS nanoparticles. Then, they were mixed with a pre-synthesized PAA solution and subjected to thermal imidization treatment to obtain a PEO-POSS/PI composite film. Finally, the PEO structure was oxidized and degraded to prepare POSS/PI thin films. With the increase of PEO-POSS content, the dielectric constant of the composite film decreased. When the addition of PEO-POSS reached 10 wt.%, the dielectric constant of POSS/PI decreased to 2.25 at 1 MHz while retaining its thermal stability and mechanical properties. Luo et al. [74] manufactured a reversible cross-linked composite by functionalizing POSS with maleimide and aromatic polyamide (POF) with a furan group. The Diels–Alder reaction between maleimide and furan groups allowed the facile cross-linking of POF by POSS, thus significantly improving the thermal and mechanical properties of POSS/POF composites. It was found that with the increase of POSS content, the dielectric constant of the composite material significantly dropped from 4.25 for pure POF to 2.25 for POF with a 0.2 molar ratio of maleimide to furan groups at 1 MHz, and the dielectric loss also decreased from 0.029 to 0.018, correspondingly. Revathi et al. [75] successfully synthesized a novel imidazole nuclear asymmetric diamine monomer (4,4-(4,5-diphenylimidazol-1,2-diyl) diphenylamine) and employed it as a precursor to prepare PI. In addition, different mass ratios of octaaminophenyl silsesquioxane (NH_2_-POSS) were used to modify PI, constructing POSS/PI composites. The results showed that as the mass fraction of NH_2_-POSS increased, the dielectric constant of the composite material decreased. At 1 MHz, the dielectric constant of 10% POSS/PI composite reached the lowest value of 2.1. Chen et al. [76] self-assembled an amphiphilic alternating copolymer with hydrophobic POSS segments and hydrophilic anhydride segments into nanoscale POSS aggregates with an average diameter of 129 nm (Figure 7a), which was incorporated into FPI to construct the POSS/FPI composite films. The dielectric constant of POSS/FPI composite films with 2.8 wt.% amphiphilic alternating copolymer significantly decreased to 2.09 from 3.14 for pure FPI at 10 MHz, as shown in Figure 7b. The internal sub-nanometric cavities of POSS and the increased free volume of POSS/PI composite films should be responsible for the decrease in the dielectric constant.

Zhang et al. [77] combined an intrinsically optically transparent PI film matrix, poly [4,4′-(hexaflfluoroisopropylidene)diphthalic anhydride-co-2,2-bis(4-(4-aminophenoxy)phenyl)hexaflfluoropropane] (6FPI), with a nanocage trisilanolphenyl polyhedral oligomeric silsesquioxane (TSP-POSS) additive to construct POSS/PI films, which manifested the dielectric constant and dielectric loss values as low as 2.52 and 0.006 at 1 MHz, respectively. Interestingly, the transparent PI composite films displayed enhanced atomic oxygen-resistant properties, showing a high prospect for antenna substrates in aerospace applications. Chen et al. [78] employed trifluoropropyl POSS to modify PI film and the dielectric constant of POSS/PI with 2.0 wt.% POSS decreased to 2.47 from 3.17 (pure PI) at 10 MHz and the corresponding dielectric loss was reduced to 0.0089 from 0.011 due to the hollow structures in the POSS molecule and the existence of fluorine substituents. He et al. [79] prepared POSS containing a hierarchical porous structure by the self-assembly of water droplets and utilized it as a coating for the flat PI film. Due to the hierarchical porous coating and the fluorine-containing groups, when the content of POSS changed from 10% to 50%, obvious declines were found in the dielectric constant from 3.36 for PI film to 2.42–2.75 at 1 MHz. In addition, the stability of dielectric properties in a humid environment was confirmed by only a 2.94–4.83% increase in dielectric constant after exposure to high humidity for more than 25 h. The same group [80] further constructed a sandwich-type composite film by coating both sides of flat FPI film with POSS containing a hierarchical porous structure using the microemulsion method, in which water droplets were used as the template for pore patterning. Figure 7c–h displayed the morphologies of D-FPI and D-POSS-PIs containing different contents of POSS, showing a macroporous structure (D: porous structure on both sides; S: porous structure on one side.). As shown in Figure 7i,j, by varying the POSS content from 10% to 50%, a low dielectric constant of 2.28–2.42 and dielectric loss of 0.005–0.009 were achieved. The decrease in dielectric constant with the incorporation of POSS is due to the pores and large free volume introduced by POSS that could weaken the dipole–dipole interaction of polymer chains in the composite films, the low polarity vinyl groups on POSS and the increased interfacial area that led to the strong self-polarization-induced dielectric confinement effect.

Continued efforts have been devoted to further lowering the dielectric constant by constructing ternary hybrid materials via integrating SiO_2_, POSS and PI due to their synergy effect. Zhao et al. [81] utilized the silane agent 2-(3,4-epoxycyclohexyl)ethyl-trimethoxysilane as a chemical bridge to graft POSS onto the surface of functionalized mesoporous silica MCM-41 and incorporated the POSS functionalized MCM-41 into a quaternary copolymerized FPI synthesized via the polycondensation reaction of 2,2-bis[4-(4-aminophenoxy)phenyl]-hexafluoropropanane, 4,4′-(9-fluorenylidene)dianiline, 4,4-(4,4-Isopropylidenediphenoxy)bis(phthalic anhydride) and (5,5′-biisobenzofuran)-1,1′,3,3′-tetraone. The SiO_2_/POSS/PI composites exhibited good hydrophobicity. With the addition of 3 wt.% SiO_2_/POSS, the PI composite manifested an ultralow dielectric constant of 1.88 and low dielectric loss of 0.01 at 1 MHz, which was ascribed to the mesoporous structure of MCM-41 and the restriction of polarization in the bonded region.

Although the introduction of POSS fillers can significantly improve the comprehensive performance of composite materials, on the one hand, the purification process of POSS is complex and the yield is low, making it difficult to produce on a large scale. On the other hand, because each vertex of POSS has the same reaction activity, most of the works on POSS employed modified POSS as the crosslinking point to form a cross-linking network, which leads to an increase in the viscosity of the entire system and a decrease in film forming ability. Moreover, the modification of POSS generally requires the introduction of alkyl chains, which will reduce the thermal stability of the material. The above-mentioned shortcomings limit the application of POSS in low k PI thin films.

## 7. Hyperbranched Polysiloxane/PI Composite Materials

In recent years, HBPSi, similar to the traditional POSS, shows good comprehensive performance. Due to its simple synthesis and low cost, it has been widely used in low dielectric, adhesive, flame retardant, antigenic oxygen and other fields [82]. The interchain packing density could be limited due to the huge steric hindrance of HBPSi, which enlarged the free volume of HBPSi-modified polymers and thus decreased the dielectric constant of corresponding materials. Moreover, due to the high bonding energy of Si–O–Si in HBPSi, the incorporation of HBPSi in the polymer matrix can effectively diminish the dielectric constant of the materials without compromising its thermal properties [83]

Li et al. [84] prepared a series of phenylethynyl-terminated PI resins by grafting amine-functionalized HBPSi to PI chains during in situ polymerization. Due to the enlarged free volume, the effect of steric hindrance and the intrinsic low dielectric constant of silsesquioxane (Si–O–Si), the dielectric constants of the composite resins greatly dropped from 3.29 to 2.19 at 10 MHz without compromising its processability and the values of dielectric loss were low, between 0.01 and 0.006. Moreover, the high cross-linking density of the HBPSi/PI composite brought about superior dielectric stability in the high-temperature region. Lian et al. [85] fabricated a series of HBPSi-based hyperbranched PI films with multiple branched structures by copolymerizing 2,4,6-triaminopyrimidine (TAP) with 4,4′-(hexaflfluoroisopropylidene) diphthalic anhydride, 4,4′-diaminodiphenyl ether and HBPSi via the two-step polymerization method. The dielectric constant of HBPSi/PI films decreased with increasing TAP fraction and reached the lowest value of 2.80 at 1 M Hz owing to the enriched free volume created by the incorporation of multiple branched structures. Moreover, HBPSi/PI possessed good mechanical properties and thermal stability.

## 8. Other PI Composite Materials

Besides the common fillers mentioned above, other fillers such as epoxy polymers and montmorillonite, etc., have also been reported to be used to synthesize porous PI hybrid membranes. Wang et al. [86] modified montmorillonite with a double-swelling agent ([2-(Dimethylamino)ethyl] triphenylphos-phonium bromide and 4,4′-oxydianiline) and used it as a filler to combine with PAA to construct PI-based composites. When the mass fraction of modified montmorillonite was 3.0 wt.%, the dielectric constant of the porous composite material could be reduced to 2.6 at 1 MHz. In addition, the glass transition temperature and storage modulus of the composite film increased. Dong et al. [87] manufactured a series of all-organic PI films with high hydrophobicity by introducing a kind of fluoroelastomer (FEM). The hydrogen bond significantly inhibited the dipolar polarization of the FEM/PI composite, which played an important role in determining the dielectric properties of the composite system. When the FEM filler content was 7 wt.%, the FEM/PI composites exhibited an extremely low dielectric constant of 1.21 at 1 MHz. Zhang et al. [88] synthesized a series of polytetrafluoroethylene (PTFE)/PI composite films by blending water-soluble PAA salts with water-based PTFE dispersion and thermal imidizaiton. It was found that the dielectric properties of PI were effectively improved by PTFE fillers and with the addition of 30 wt.% PTFE filler, the PTFE/PI composite film showed a reduced dielectric constant of 2.42 and dielectric loss of 0.0044 at 10 GHz due to the fact that the –CF_3_ group in the molecular structure not only enriched the free volume and hindered the dipole orientation process, but also largely diminished the polarization rate due to its extremely electronegative nature. Cheng et al. [89] developed a low dielectric and high adhesive fluorinated ethylene propylene (FEP/PI) composite film. Because the fluorocarbon surfactants could significantly improve the dispersion of FEP in a PI substrate, the FEP/PI composite film exhibited good mechanical properties. Moreover, the 60 wt.% FEP-filled PI composite film displayed a reduced dielectric constant of 2.69 and an ultralow dielectric loss of 0.006 at 10 GHz. Li et al. [90] synthesized three kinds of bisphenol A-containing diamine (2,2′-bis[4-(4-aminophenoxy)phenyl]propane, 2,2-bis[4-(2-methyl-4-aminophenoxy)phenyl] propane and 2,2-bis[4-(2-triflfluoro methyl-4-aminophenoxy)phenyl]propane) and they were polymerized with 4,4′-(hexaflfluoroisopropylidene)diphthalic anhydride to prepare low-dielectric PI films by thermal imidization. Crown ethers (CEs) were then introduced into the PI with different mass ratios to construct CE/PI composite films. The CE dispersed uniformly in the PI matrix offered the enhanced free volume of the PI matrix and generated a special necklace-like supramolecular structure, resulting in improved dielectric properties. The dielectric constant and dielectric loss of the composite films were remarkably reduced to 2.33 and 0.00337 at 10 GHz, respectively. Zhang et al. [91] synthesized a series of tetraamino phthalocyanine zinc (TAPcZn) micro-crosslinked PI films by the copolymerization of 3,3′,4,4′-benzophenone tetracarboxylic dianhydride and ODA. The 4.49 wt.% TAPcZn/PI film showed an ultra-low dielectric constant of 2.33 at 1 MHz and a dielectric loss of less than 0.007 due to the microbranching effect ascribed to a stronger enlarging molecular interlayer with the incorporation of TAPcZn. In addition, the composite displayed outstanding flame-retardant performance.

Furthermore, some new functions like UV-light shielding and anti-photoaging properties have been endowed on TiO_2_/PI composite nanofibers due to the introduction of TiO_2_ nanoparticles with high UV-light absorption and scattering ability, while not obviously affecting its low dielectric properties [92]. These explorations to date have widened the application field of low k PI-based composites.

## 9. Summary and Outlook

Nowadays, low k porous PI materials have made a series of fruitful research achievements, which have laid a good foundation for the development of a new generation of ultra-low k dielectric materials. This paper mainly summarizes the strategies of preparing low k PI-based composites by introducing porous SiO_2_, graphene derivatives, POMs, POSS, HBPSi, etc. Table 1 lists the dielectric constants of low k PI-based composite materials reported in the literature. It is worth noting that no matter what kind of porous nanoparticles are introduced into the PI matrix, their essence is the same, which is to build holes with a skeleton structure in the PI matrix, so as to reduce the dielectric constant of PI. However, the dielectric properties of PI-based porous composites are different due to different porous nanoparticles. From the perspective of diminishing the dielectric constant of PI, the incorporation of porous nanoparticle fillers can also reduce the dielectric constant of PI to a certain extent, but in order to meet the demands of integrated circuits for interlayer insulation materials, a higher amount of additives is required. In addition, no matter what kind of porous nanoparticle filler is used to prepare low k PI composites, there exist certain defects. For example, the purification process of POSS is complex and the yield is low, while the preparation of graphene derivatives is costly, which limits their application in low k PI films. Although the addition of a small amount of porous SiO_2_ reduces the dielectric constant of PI, it also deteriorates the mechanical properties of PI itself. If the amount of SiO_2_ added is too large, it is easy to observe an increase in dielectric constant caused by filler aggregation or filler structure collapse. Moreover, the compatibility between POMs and PI is poor, and surface modification is required to perform bulk filling.

More importantly, the inherent drawbacks of porous materials still pose a bottleneck for the use of porous low k materials in ultra-large-scale integrated circuits. Firstly, hole connecting is easy to form, and cannot achieve effective isolation of metal wires. Secondly, compared to dense materials, porous materials have poor mechanical properties and weak adhesion with the upper layer material and lower layer matrix. Therefore, in addition to further reducing their dielectric constant, it is also necessary to improve their mechanical properties and bonding properties with metal materials. Moreover, when preparing low k PI, most studies only consider diminishing the dielectric constant of PI itself, as well as improving its mechanical properties and thermal stability, ignoring the disadvantage of the high moisture absorption of PI itself. Therefore, except for continuously diminishing the dielectric constant of PI without sacrificing its thermal and mechanical properties to meet the development of microelectronics, the hygroscopicity of PI should also be reduced. For example, fluorination modification of the PI surface or matrix [105] or the introduction of hydrophobic cross-linked networks [106] can be considered to improve the hydrophobicity of the material. The final issue is that the dielectric properties of PI are closely related to temperature. In high-frequency environments, devices generate heat during operation and the temperature increases. At this time, the dielectric properties of PI materials will undergo further changes, which is a factor that cannot be ignored in the application process of PI materials. Some materials, such as porous boron nitride (BN) with high thermal conductivity were suggested as filler to construct a BN/PI composite where the heat transfer occurs easily in the composite and low dielectric constants of 2.08~3.48 at a high frequency of 1 GHz for BN content of 20–80 wt.% was also achieved [107]. However, related mentions of a PI composite with high thermal diffusivity in the existing literature are still not enough. Therefore, in the future, monomer molecular structure design, filler modification and process optimization should be further carried out for the synthesis of porous PI. On the premise of significantly improving the dielectric properties, mechanical properties, thermal stability, hydrophobicity and other properties of composite films, the practical application of PI materials can be improved, and the experimental process can be optimized to make the synthesis process of composite materials more simple and efficient, with a higher synthesis rate, and more suitable for industrial production, thus promoting the development of large-scale integrated circuit chips towards miniaturization.

## Figures and Tables

**Table 1:** Summary of low k PI-based composite materials reported in the literature.

Additive	Dielectric Constant	Frequency	Ref.
SiO_2_	2.61	1 MHz	[43]
	2.45	1 kHz	[44]
	2.61	10 MHz	[45]
	2.6	1 MHz	[46]
	2.5	1 MHz	[47]
	2.9	1 MHz	[48]
	2.2	1 kHz	[50]
	2.58	28 GHz	[51]
	2.09	1 kHz	[52]
	2.24	1 kHz	[53]
	1.62	28 GHz	[54]
	2.65	1 MHz	[93]
	1.81	1 MHz	[94]
Glass	2.21	1 MHz	[95]
GO	2.58	10 MHz	[58]
	2.21	10 MHz	[59]
	2.0	1 GHz	[56]
	1.9	1 GHz	[57]
	2.58	10 MHz	[58]
	2.21	10 MHz	[59]
	2.96	1 MHz	[60]
	2.1	1 MHz	[96]
rGO	1.93	1 MHz	[61]
FG	2.1	1 MHz	[62]
	1.77	1 kHz	[63]
	1.56	10 kHz	[64]
	1.92	10 kHz	[65]
	2.65	1 MHz	[97]
POM	2.05	1 MHz	[66]
POSS	2.4	1 MHz	[69]
	2.53	-	[70]
	2.30	100 kHz	[71]
	2.12	100 kHz	[72]
	2.25	1 MHz	[73]
	2.25	1 MHz	[74]
	2.1	1 MHz	[75]
	2.09	10 MHz	[76]
	2.52	1 MHz	[77]
	2.47	10 MHz	[78]
	2.42	1 MHz	[79]
	2.28	1 MHz	[80]
	2.21	1 MHz	[98]
	2.6	1 MHz	[99]
	2.68	1 MHz	[100]
	2.6	1 MHz	[101]
HBPSi	2.19	10 MHz	[83]
	2.8	1 MHz	[84]
SiO_2_/POSS/PI	1.88	1 MHz	[85]
Montmorillonite	2.6	1 MHz	[86]
FEM	1.21	1 MHz	[87]
PTFE	2.42	10 GHz	[88]
FEP	2.69	10 GHz	[89]
CE	2.33	10 GHz	[90]
	2.74	1 MHz	[102]
Polypropylsilsesquioxane/CE	2.71	1 MHz	[103]
TAPcZn	2.33	1 MHz	[91]
Fluorinated nanocarbon	2.75	1 MHz	[104]

## Data Availability

Not applicable.
